# Oocyte-specific disruption of adrenomedullin 2 gene enhances ovarian follicle growth after superovulation

**DOI:** 10.3389/fendo.2022.1047498

**Published:** 2022-11-14

**Authors:** Chia Lin Chang, Wei-Che Lo, Ta-Hsien Lee, Jia-Yi Sung, Yen Ju Sung

**Affiliations:** Department of Obstetrics and Gynecology, Chang Gung Memorial Hospital Linkou Medical Center, Chang Gung University, Taoyuan, Taiwan

**Keywords:** ovary, oocyte, adrenomedullin, ADM2, CGRP, transgenic

## Abstract

**Background:**

Adrenomedullin 2 (ADM2), adrenomedullin (ADM), and calcitonin gene-related peptides (α- and β-CGRPs) signal through heterodimeric calcitonin receptor-like receptor/receptor activity-modifying protein 1, 2 and 3 (CLR/RAMP1, 2 and 3) complexes. These peptides are important regulators of neurotransmission, vasotone, cardiovascular development, and metabolic homeostasis. In rodents, ADM is essential for regulating embryo implantation, fetal–placental development, and hemodynamic adaptation during pregnancy. On the other hand, ADM2 was shown to affect vascular lumen enlargement, and cumulus cell-oocyte complex (COC) communication in rodent and bovine ovarian follicles. To investigate whether oocyte-derived ADM2 plays a physiological role in regulating ovarian folliculogenesis, we generated mice with oocyte-specific disruption of the Adm2 gene using a LoxP-flanked Adm2 transgene (Adm2 loxP/loxP) and crossed them with Zp3-Cre mice which carry a zona pellucida 3 (Zp3) promoter-Cre recombinase transgene.

**Results:**

While heterozygous Adm2 +/-/Zp3-Cre and homozygous Adm2 -/-/Zp3-Cre mice were fertile, Adm2 disruption in oocytes significantly increased the number of ovulated oocytes following a superovulation treatment. Oocyte-specific Adm2 disruption also significantly impaired the developmental capacity of fertilized eggs and decreased the size of the corpus luteum following superovulation, perhaps due to a reduction of ovarian cyclin D2-associated signaling.

**Conclusions:**

The disruption of intrafollicular ADM2 signaling leads to follicular dysfunction. These data suggested that oocyte-derived ADM2 plays a facilitative role in the regulation of hormonal response and follicle growth independent of the closely related ADM and CGRP peptides, albeit in a subtle manner.

## Introduction

Adrenomedullin 2/intermedin (ADM2/IMD) belongs to a peptide family that includes adrenomedullin (ADM), calcitonin gene-related peptides (α- and β-CGRPs), calcitonin, and amylin (1–5). ADM, ADM2, and CGRPs are structurally similar and signal through receptor complexes consisting of calcitonin receptor-like receptor (CLR) and one of the three receptor activity-modifying proteins (RAMP1, 2 and 3) (1, 2, 5–7). While CGRPs and ADM mainly signal through CLR/RAMP1 and CLR/RAMP2, respectively (6, 8), ADM2 is a mild agonist with no distinct preference for the three CLR/RAMP receptors (1).

CGRPs are important for the regulation of nociception, hyperalgesia, and allodynia (9, 10), and excessive CGRP release is associated with migraine and joint pain (11, 12). On the other hand, ADM is essential for the regulation of vasotone and endothelial barrier integrity as well as the proliferation of blood and lymphatic endothelial cells (5, 13–31). Mice deficient in *Adm*, *Clr*, or *Ramp2* gene die *in utero* with cardiovascular abnormalities. Heterozygous *Adm* ^+/-^ mice are hypertensive/obese and have increased mortality under stress conditions (32–34). On the other hand, *Adm2* was recently shown to be important for the regulation of vascular lumen enlargement in mice (35). Of interest, the ADM2 transcript was shown to be preferentially expressed in rodent and human oocytes (1, 36). In addition, we have shown that ADM2 facilitates cell-cell interactions in cumulus-oocyte complexes (COCs) by improving the expression of cell cycle progression genes such as cyclin D2 (37, 38). Likewise, in bovine follicles, ADM2 was shown to act as a secretory factor controlling COCs conformation (39) and improve oocyte competence and embryo quality (40). Only embryos from COCs treated with ADM2 could develop into stage-6 grade I blastocysts while blockage of ADM2 signaling inhibited normal COC formation. However, whether the endogenous oocyte-derived ADM2 is essential for the regulation of folliculogenesis remains to be vetted. The various roles attributed to oocyte-derived ADM2 may be mediated by endogenous ADM or CGRPs which act through the same receptors.

**Figure 1:**
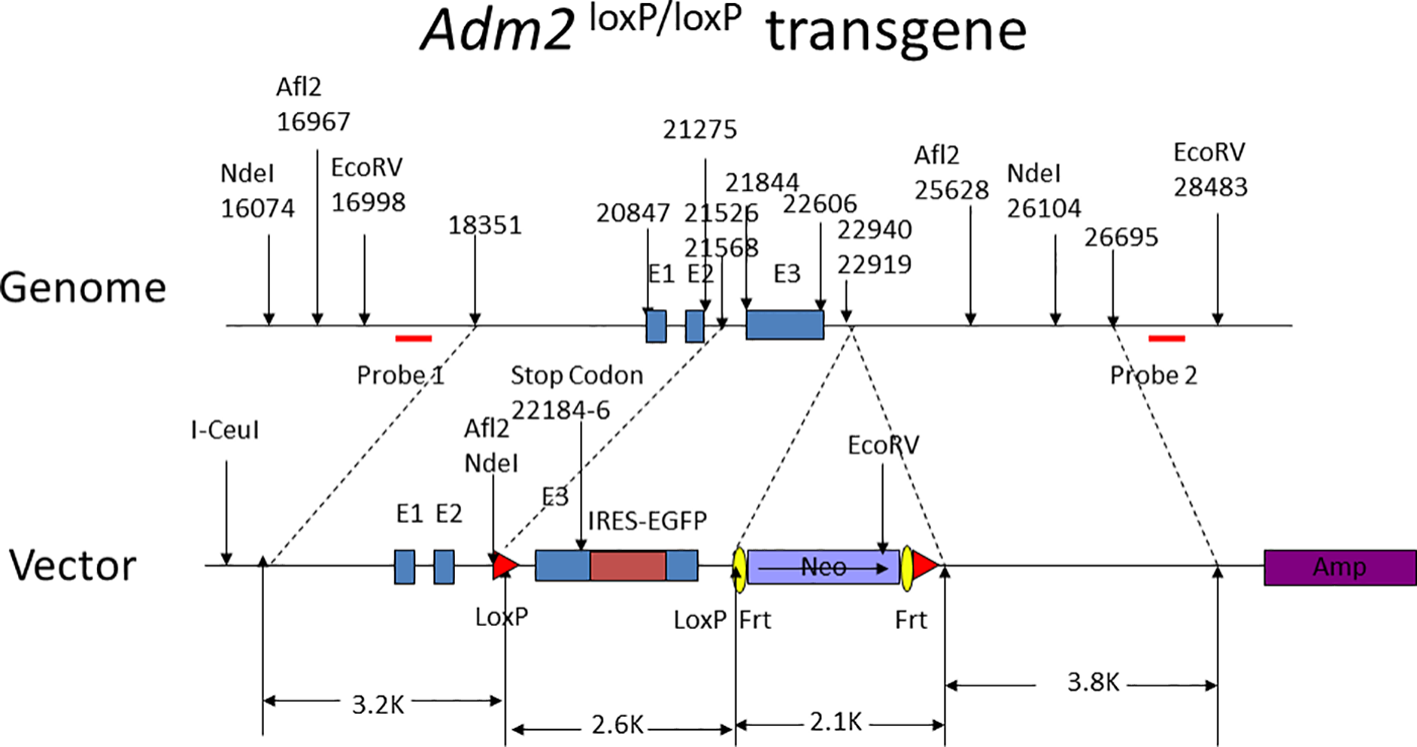
Design of the *Adm2 ^loxP/loxP^* transgene. The nucleotide numbers indicate the relative positions of subcloning sites, exons, and restrictive enzyme cleavage sites. Probes 1 and 2 for the detection of the transgene are indicated by red horizontal bars. The Neo and Amp selection genes are labeled. The exon I, II, and III of the *Adm2* gene are labeled as E1, E2, and E3, respectively. The transgene also has an EGFP tag for transgene detection. The I-Ceul site was used to linearize the plasmid.

To investigate the physiological role of oocyte-derived ADM2, we generated mice with oocyte-specific disruption of the *Adm2* gene using a LoxP-flanked *Adm2* transgene (*Adm2 ^loxP/loxP^*; **Figure 1**) and a zona pellucida 3 (*Zp3*) promoter-Cre recombinase transgene. Analysis of the reproductive physiology of these mice showed that intrafollicular ADM2 signaling plays a niche role in the regulation of folliculogenesis independent of the closely related ADM and CGRPs (41). Further studies of the role of ADM2 signaling during folliculogenesis may facilitate our ability to improve follicle development in infertile patients.

## Materials and methods

### Construction of the Adm2 ^loxP/loxP^ transgene

The *Adm2* ^loxP/loxP^ transgene, as illustrated in **Figure 1**, was engineered to have the *Adm2* exon 3 flanked by a pair of LoxP sequences. The transgene was electroporated into murine embryonic stem (ES) cells, and positive clones were identified by medium selection and PCR amplification analyses. Positive ES clones with correct homologous recombination were identified by Southern blotting of genomic DNAs.

ES cell pellets were routinely dissolved in 0.5 ml lysis buffer in 24-well plates, mixed with 10 μl Proteinase K (10 mg/ml), and digested at 55C for 12 hr. The DNA solution was extracted with phenol twice, followed by extraction with chloroform. DNA samples were precipitated with isopropanol. After centrifugation, the DNA pellets were washed with 70% alcohol, dried, and dissolved in the TE buffer. The selected clone was injected into blastocysts from the C57BL/6 strain mice to generate chimeric embryos at the transgenic mouse core facility of the National Taiwan University.

### Generation and characterization of transgenic mice with Adm2 ^loxP/loxP^ and Zp3-Cre transgenes

Chimeric mice were bred to obtain heterozygous lines, and homozygous animals were generated *via* selected breeding of heterozygous animals. The genotypes of animals were screened by PCR amplification of genomic DNA using probes that were designed to differentiate the *Adm2 ^loxP/loxP^* transgene from the wild-type sequence (**Figure 1**). All animals were managed in full compliance with the requirements of the Animal Welfare Act and in accord with the guidelines of the Committee on Care and Use of Laboratory Animals.

In transgenic mice, the *Zp3*-Cre transgene is specifically expressed in oocytes of developing follicles starting on day 5 after birth (42), and has been used to investigate the role of oocyte-expressed gap junction *Cx43* (43), *Mgat1* (44), terminal galactose or N-acetylglucosamine (45, 46), beta-catenin (47), *Msy2* (48), furin (49), *Cdx2* (50), and focal adhesion kinase (51). By crossing the *Adm2 ^loxP/loxP^* transgenic mice with *Zp3*-Cre mice (C57BL/6-Tg(Zp3-cre)93Knw/J; https://www.jax.org/strain/003651), we obtained mice with oocyte-specific *Adm2* gene disruption prior to the first meiotic division (i.e., heterozygous *Adm2* ^+/-^/*Zp3*-Cre and homozygous *Adm2* ^-/-^ */Zp3*-Cre mice).

### Breeding and characterization of the Zp3-Cre/Adm2 ^loxP/loxP^ transgenic mice

Mice were bred and housed at the Transgenic Mouse Models Core Facility, National Research Program for Genomic Medicine, Taipei. Mice were maintained in a controlled environment of 20-22C under SPF conditions, with a 12/12 hr light/dark cycle, and a 50-70% humidity. Both *Adm2* ^loxP/loxP^ and *Zp3*-Cre mice had a C57BL/6 genomic background, and animals with the *Adm2^+/+^*/*Zp3*-Cre (wild-type), *Adm2^+/-^*/*Zp3*-Cre (heterozygous), or *Adm2^-/-^*/*Zp3*-Cre (homozygous) genotypes were retained for functional characterization. Specific TaqMan primers that target *Adm2* exon 3 sequences were used to differentiate the wild-type and transgene transcripts. Probes were labeled with the reporter fluorochrome 6-carboxyfluorescein (FAM) at the 5’-end and the quencher fluorochrome 6-carboxy-tetramethyl-rhodamine (TAMRA) at the 3’-end. Real-time PCR was conducted using a LightCycler^®^ 480 System (F. Hoffmann-La Roche Ltd, Basel, Switzerland).

### Analysis of morphogenesis and gene expression

Once the homozygous *Adm2* ^loxP/loxP^ mice were established, we expanded the colony for the analysis of general physiology. Because *Adm2* is expressed in diverse vascular beds, major internal organs were visually examined for signs of abnormalities during the autopsy. To evaluate the expression of *Adm2* in tissues, select organ samples, including kidney and ovary, were collected for qPCR analysis. Mouse *Adm2* cDNA and the transgene construct were used as the controls.

### Fertility testing

To study the effects of the transgene on fertility, 7- to 8-week-old female wild-type, heterozygous, and homozygous mice were mated with 10- to 12-week-old males over a 20-week period. The number of pups and litter was recorded.

### Superovulation, fertilization, and embryo development *in vitro*

To study the effect of *Adm2* deficiency on ovarian folliculogenesis and ovulation under pharmacological conditions, immature 26-day-old female mice were intraperitoneally injected with 5 IU of pregnant mare’s serum gonadotropin (PMSG; Sigma-Aldrich^®^ Brand, Merck KGaA, Darmstadt, Germany) to induce follicular growth. Animals were then injected with 5 IU human chorionic gonadotropin (hCG) at 44 hr after PMSG stimulation to induce ovulation, followed by mating with wild-type males. At 46 hr after hCG injection, embryos (i.e., Embryo 1.5 day) and unfertilized eggs were retrieved from oviducts surgically and cultured with M16/PBS medium (Sigma-Aldrich^®^ Brand, Merck KGaA, Darmstadt, Germany) in a 5% CO_2_ incubator at 37C with 95% humidity for 2 days. The numbers of multi-cell embryos and blastocysts were recorded.

### Histology and immunohistochemistry of ovarian sections

To evaluate follicle development following gonadotropin stimulation, ovarian tissues collected after PMSG or superovulation treatment were fixed in Bouin’s solution and embedded in paraffin (52), and serially sectioned at 5 μm thickness. Sections were stained with H&E for morphological evaluation. Ovarian follicles at different developmental stages were classified according to their size and their tertiary topology: primary, oocytes were covered with a single layer of cuboidal granulosa cells; secondary, with multiple layers of granulosa cells, but without an antrum (100-140 μm); early antral, 140–200 μm follicles with an antrum; large antral, >200 μm in diameter and with a distinct cumulus cell layer surrounding the oocyte. The corpus luteum was recognized based on its distinctive histological characteristics. The size of an individual corpus luteum was quantified by calculating the sum of length and width divided by 2. The length was defined as the longest diameter of a corpus luteum, and the width was the measurement perpendicular to that of the length.

For immunohistochemical analysis of cyclin D2 protein, which normally promotes G1 progression by activating cyclin-dependent kinase-4 in growing follicles, we obtained ovarian tissues at 44 hr after PMSG stimulation. Antigen retrieval was performed in 0.01 M sodium citrate in a microwave for 10 min. After quenching with 3% H_2_O_2_, ovarian sections were washed in Tris-buffered saline (TBS) and incubated with a primary antibody against cyclin D2 (AB3087; Abcam Plc., Cambridge, UK) at 4C overnight, followed by a reaction with secondary antibodies. Nonspecific binding was blocked by incubating slides in TBS containing 0.1% Triton X-100 and 10% goat serum. An avidin-biotin peroxidase complex method was used to visualize the antigen antibody complex with the Dako Liquid DAB Chromogen System (Sigma-Aldrich^®^ Brand, Merck KGaA, Darmstadt, Germany) (38). The signal intensity for cyclin D2 was captured with a Zeiss microscope system. The immunohistochemical staining was quantified using the HistoQuest and Image J software (StrataQuest analysis Apps, TissueGnostics GmbH, Vienna, Austria; Western Pacific Division, Taipei, Taiwan), and the relative signal of individual ovarian sections was calculated by subtracting the observed signal with negative control (i.e., without primary antibody treatment).

### Statistical analysis

Statistical analysis was conducted by one-way ANOVA or *t*-test, and the significance was accepted at *p* < 0.01.

## Results

### Disruption of the Adm2 gene in oocytes has no obvious effects on fecundity

**Figure 2:**
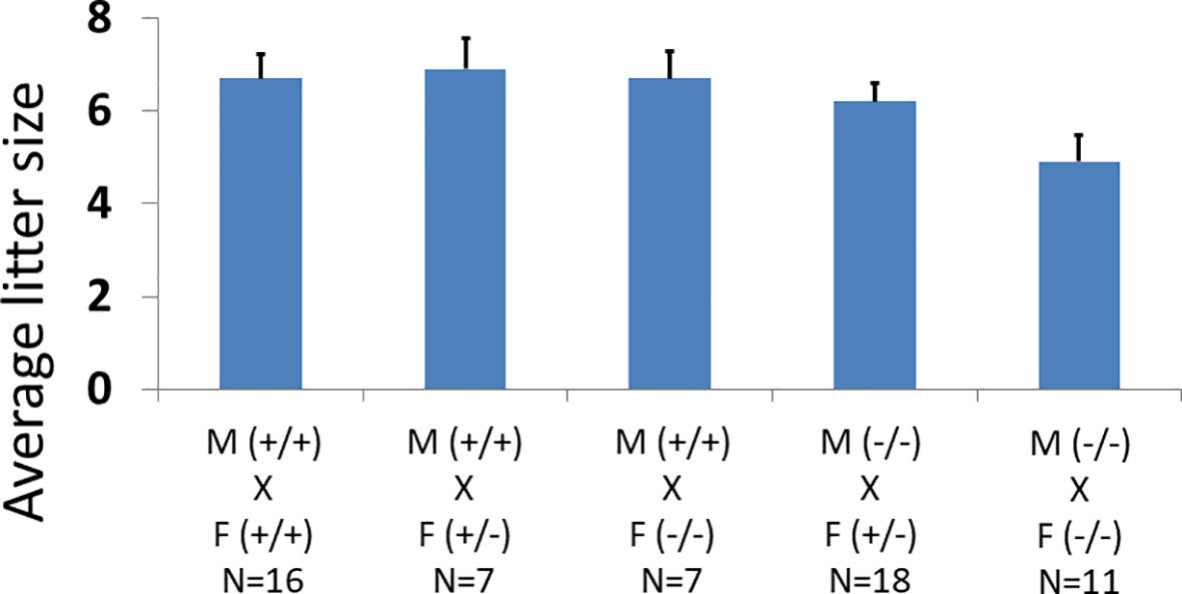
Effects of oocyte-specific *Adm2* disruption on the fecundity of female mice. The average litter size of wild-type (*Adm2^++^*/*Zp3*-Cre), heterozygous (*Adm2^+/-^*/*Zp3*-Cre), and homozygous (*Adm2^-/-^/Zp3*-Cre) mice are represented by vertical bars (mean ± SEM). The crosses included those between wild-type males (M, +/+), and wild-type (F, +/+, N=16), heterozygous (F, +/-, N=7) or homozygous (F, -/-, N=7) females as well as those between homozygous males (M, -/-), and heterozygous (F, +/-, N=18) or homozygous (F, -/-, N=11) females. The litter size ranged from 4.9 ± 0.56 in crosses between homozygous males and homozygous females to 6.9 ± 0.67 in crosses between wild-type males and heterozygous females.

We generated mice with conditional knockout of *Adm2* in developing oocytes using an *Adm2 ^loxP/loxP^* transgene and the *Zp3*-Cre transgenic mice (**Figure 1**; **Supplementary Figure 1**) (42). Analysis of breeding records showed wild-type (*Adm2^+/+^/Zp3*-Cre), heterozygous (*Adm2^+/-^/Zp3*-Cre), and homozygous (*Adm2^-/-^/Zp3*-Cre) mice were fertile. The fecundity was similar among genotype groups (**Figure 2**). Autopsies of adult mice showed there is no gross change of major organs in transgenic mice. Analysis of *Adm2* mRNA expression in ovaries and kidneys showed that the wild-type *Adm2* transcript is absent in homozygous animals (**Supplementary Figure 2**).

### Disruption of the Adm2 gene in oocytes enhances ovarian follicle growth following gonadotropin stimulation

While *Adm2* disruption in oocytes did not have an obvious effect on fecundity, we reasoned the function of oocyte-derived ADM2 may be masked by endogenous ADM or CGRP peptides under physiological conditions. The potential role of oocyte-derived ADM2 may be revealed when animals are subjected to pharmacological stimulation. Accordingly, we induced synchronized ovulation in female mice using a standard superovulation regimen and mated them with wild-type males (53).

**Figure 3:**
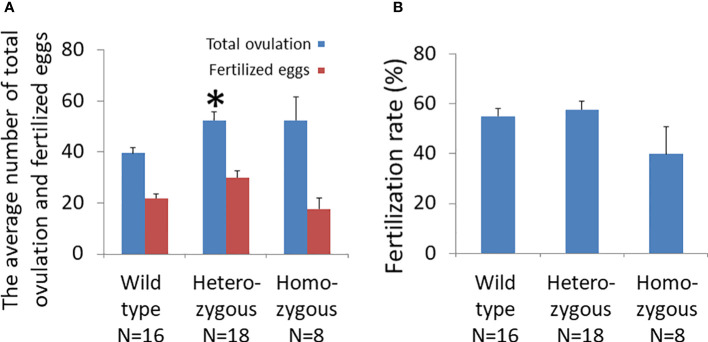
Effects of oocyte-specific *Adm2* disruption on ovulation rate following superovulation. **(A)** The numbers of ovulated oocytes and fertilized eggs of wild-type (*Adm2^+/+^/Zp3*-Cre, N=16), heterozygous (*Adm2^+/-^/Zp3*-Cre, N=18), and homozygous (*Adm2 ^-/-^/Zp3*-Cre, N=8) mice are represented by blue and red vertical bars, respectively (mean ± SEM). Ovulated oocytes were collected at 46 hr after hCG injection. The homozygous animals had a higher number of ovulated oocytes compared to wild-type mice, and the difference was at the border of significance (*p* = .08). **(B)** The fertilization rate of ovulated eggs (mean ± SEM). The fertilization rate ranged from 39.8 ± 11.0% in homozygous females to 57.4 ± 3.4% in heterozygous females. *, significantly different from the wild-type at *p <*0.01.

Gonadotropin treatment led to superovulation in all animals (**Figure 3A**). The number of unfertilized eggs and multiple-cell embryos in oviducts of wild-type, heterozygous, and homozygous mice were 39.6 ± 2.1, 52.4 ± 3.2, and 52.5 ± 8.5, respectively. The number of ovulated oocytes of heterozygous animals (N=18) was significantly higher than that of wild-type mice (N=16). Likewise, the homozygous animals (N=8) had a higher number of ovulated oocytes compared to wild-type animals, and the difference was at the border of significance (*p* = .08).

Analysis of the developmental status of fertilized eggs showed that the number of 2- and 4-cell embryos from heterozygous animals (30.1 ± 2.6) was significantly higher than those of homozygous (17.6± 4.0) and wild-type (21.9 ± 1.7) animals (**Figure 3A**). However, the fertilization rate of oocytes from homozygous mice (39.8 ± 11.0%) was lower than were those of wild-type (55.1 ± 3.0%) and heterozygous (57.7 ± 3.4%) animals (**Figure 3B**).

### Oocytes of homozygous mice had impaired developmental capacity

**Figure 4:**
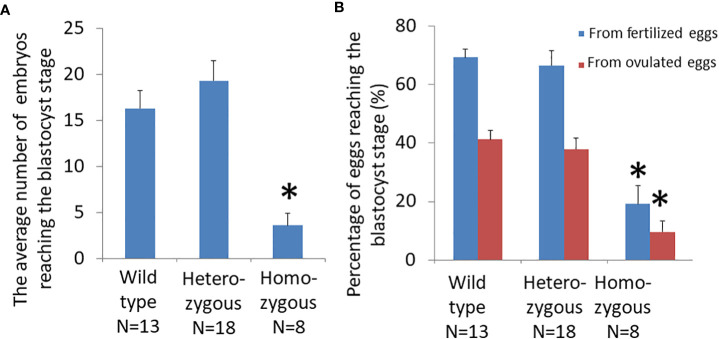
Effects of oocyte-specific *Adm2* disruption on oocyte quality. **(A)** The number of blastocysts on E3.5 day from wild-type (*Adm2^++^/Zp3*-Cre, N=13), heterozygous (*Adm2^+/-^/Zp3*-Cre, N=18), and homozygous (*Adm2^-/-^/Zp3*-Cre, N=8) mice. **(B)** The percentage of ovulated eggs and fertilized eggs that developed to the blastocyst stage following culture *in vitro* for 48 hr (mean ± SEM). *, significantly different from the wild-type or heterozygous groups at *p <*0.01.

Analysis of cultured embryos at E3.5 day showed that the average number of fertilized eggs that reached the blastocyst stage was significantly different among genotypes (**Figure 4A**; wild type: 16.3 ± 1.9; heterozygous: 19.3 ± 2.2; and homozygous: 3.6 ± 1.3). The number of blastocysts from homozygous mice was significantly lower than those of heterozygous or wild-type animals. Most fertilized eggs from homozygous animals were arrested at early stages of development.

The ratio of ovulated eggs that reached the blastocyst stage for wild-type, heterozygous, and homozygous animals was 41.1 ± 3.0%, 37.9 ± 3.7%, and 9.6 ± 3.7%, respectively. The ratio of fertilized eggs that reached the blastocyst stage for wild-type, heterozygous, and homozygous animals was 69.3 ± 2.9%, 66.4 ± 5.2%, and 19.2 ± 6.1%, respectively. Therefore, the blastocyst formation rate of homozygous mice was significantly lower than those of wild-type and heterozygous mice (**Figure 4B**).

### Adm2 disruption in oocytes reduces cyclin D2 expression in tertiary follicles and the size of corpus luteum

**Figure 5:**
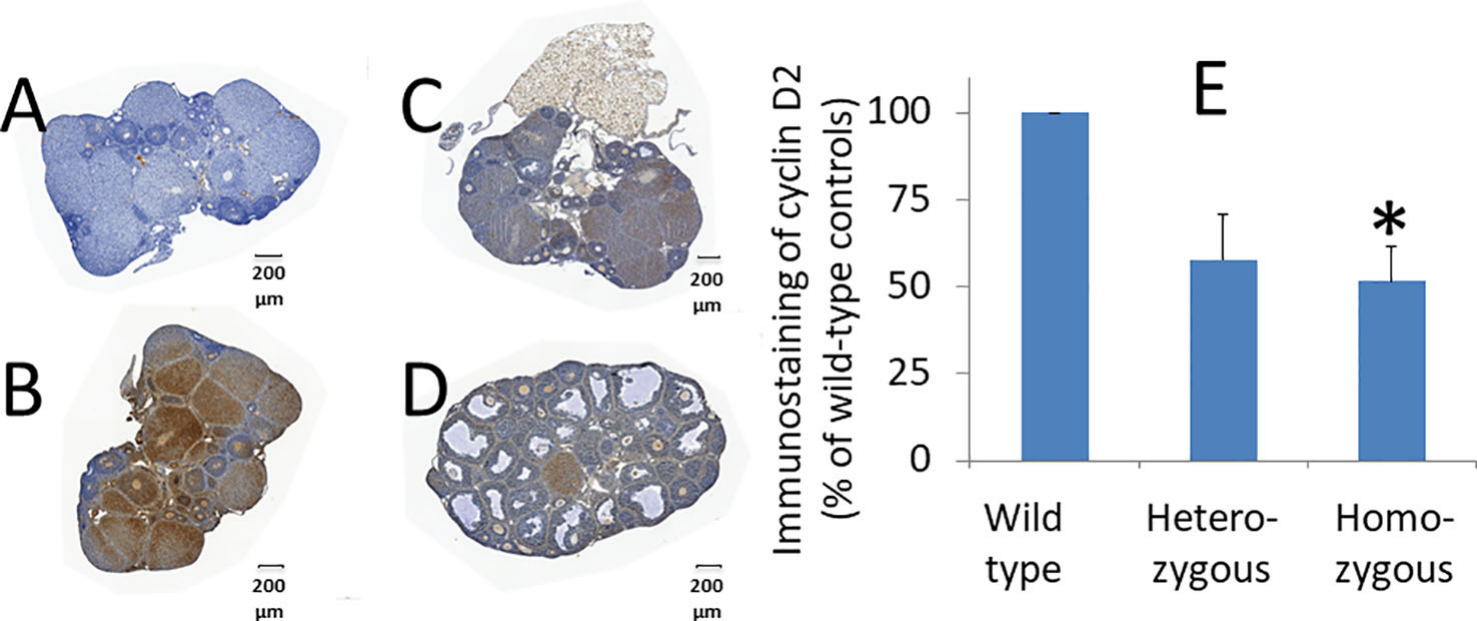
Effects of oocyte-specific *Adm2* disruption on cyclin D2 expression in growing follicles. The expression of immunoreactive cyclin D2 in ovaries of wild-type (**A**, negative control and **B**, positive control), heterozygous **(C)**, and homozygous **(D)** mice at 44 hr after PMSG treatment (mean ± SEM). The immunoreactive cyclin D2 was detected using an anti-cyclin D2 antibody (Santa Cruz Inc.). Representative sections were shown at 100x magnification. **(E)** Immunohistochemical DAB (3,3′-diaminobenzidine) staining of representative sections was quantified with the HistoQuest software. The relative density of DAB staining is represented by vertical bars. The staining in wild-type ovaries was arbitrarily set as 100% (N =3). The difference in DAB staining between wild-type and heterozygous animals was at the border of significance (*p* = 0.03). *, significantly different from the wild-type group at *p <*0.01.

To investigate how *Adm2* disruption may enhance folliculogenesis/oogenesis, we analyzed cyclin D2 expression in ovaries after treatment with pregnant mare’s serum gonadotropin (PMSG) (38). Immunohistochemical analysis of ovarian sections of mice that were primed with PMSG for 44 hr showed that cyclin D2 is abundantly expressed in large follicles of wild-type animals (**Figures 5A–D**). Quantitative analysis of DAB staining showed cyclin D2 expression is lower in heterozygous and homozygous animals, and the difference between wild-type and homozygous mice is significant (**Figure 5E**). The difference in DAB staining between wild-type and heterozygous animals was at the border of significance (*p* = 0.03).

**Figure 6:**
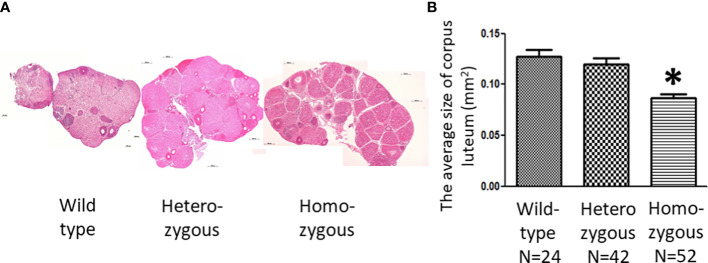
Effects of oocyte-specific *Adm2* disruption on corpus luteum following synchronized ovulation. **(A)** Histological sections of representative ovaries following superovulation. All ovaries contained a large number of corpus luteum, and the relative size is indicated by the horizontal scale bars. **(B)** The average size of corpus luteum of wild-type (*Adm2^+/+^/Zp3*-Cre), heterozygous (*Adm2^+/-^*/*Zp3*-Cre), and homozygous (*Adm2^-/-^*/*Zp3*-Cre) mice following superovulation (mean ± SEM). The size of the corpus luteum was individually determined by the HistoQuest and Image J software. The average size (mm^2^) of all corpora lutea within two representative sections of an ovary is represented by vertical bars. *, significantly different from the wild-type group at *p <*0.01.

In addition, we noticed that the ovaries of homozygous animals exhibit distinct morphological characteristics after superovulation (**Figure 6A**). Histological analysis showed the average size of corpora lutea of homozygous animals was significantly smaller than that of wild-type animals (**Figure 6B**).

## Discussion

Studies of transgenic mice showed that oocyte-specific *Adm2* disruption enhances ovarian hormonal responses and follicle growth following superovulation treatment. However, the developmental capacity of ovulated eggs and the size of the corpus luteum of homozygous animals were significantly reduced. Together, these data suggested that (1) oocyte-derived ADM2 plays a niche role in regulating ovarian folliculogenesis independent of ADM- and CGRP-mediated CLR/RAMP signaling, and (2) disruption of intrafollicular ADM2 signaling enhances hormonal response to gonadotropins and causes follicular growth dysfunction.

CGRP-CLR/RAMP1 signaling is important for the regulation of nociception and hyperalgesia (9, 10), whereas ADM-CLR/RAMP2 signaling is essential for maintaining endothelial barrier integrity as well as blood and lymphatic vessel development (5, 13–23, 54). On the other hand, CLR/RAMP3 signaling participates in the regulation of postmenopausal obesity and metabolic disorders as well as cardiac lymphatic vessel development (54, 55). Because ADM2 shares receptors with ADM and CGRPs and because ADM2 has a relatively mild receptor-activation activity, the physiological role of ADM2 is less understood. Using transgenic mice, ADM2 was recently shown to be important for the regulation of vascular lumen enlargement (35). In the ovary, ADM and CGRPs are mainly expressed in growing follicles and corpora lutea, and nerve endings, respectively (1, 56–60). Like ADM, ADM2 expression is localized in granulosa cells, blood vessels, cumulous oophorus, and corpus luteum (38, 61). Importantly, *Adm2* is also highly expressed in oocytes (1, 36), and we have shown that ADM2 is important for maintaining the integrity of cumulus-oocyte complex (COC) *in vitro* and normal cyclin D2 expression in follicles (38). Consistent with these observations, blockage of ADM2 signaling impaired follicle growth *in vitro* and ovulation in gonadotropin-primed rats, perhaps due to the inhibition of estradiol-mediated signaling pathways (39, 61). Despite these observations, whether oocyte-derived ADM2 is essential for normal folliculogenesis physiologically remains to be determined.

The observation that oocyte-specific *Adm2* disruption did not affect fertility under physiological conditions is consistent with a recent report of mice with global deficiency of *Adm2* (35). The lack of effects on fecundity could be attributed to the presence of redundant free-flowing ADM or CGRPs *in vivo*. Importantly, we showed that ADM2’s role in coordinating ovarian follicle growth can be revealed under pharmacological conditions. While *Adm2* disruption in oocytes did not lead to obvious abnormality, female transgenic mice produced significantly more oocytes following stimulation with exogenous gonadotropins. In addition, we found that fertilized eggs from homozygous mice exhibit impaired developmental capacity *in vitro*. Although earlier studies have shown that ADM2 facilitates follicle growth and COC formation *in vitro* (38, 39, 61), the present study actually found that oocyte-specific *Adm2* disruption enhances the ovulation rate after gonadotropin treatment. These results suggested the oocyte-derived ADM2 plays a more complex role in folliculogenesis and may act to limit the number of growing follicles that can reach maturity after a gonadotropin surge *in vivo* independent of the closely related ADM and CGRPs. Consistent with this hypothesis, earlier studies of IVF patients have reported that (1) follicular fluid ADM level is inversely correlated with the total number of oocytes retrieved from patients (62) and (2) follicular fluid ADM2 level is significantly higher in non-responding IVF patients compared to those of responsive groups (63). Therefore, a balanced intrafollicular ADM2 signaling may be important for determining the number of follicles that can reach final maturation. In addition to *Adm2*, conditional deletion of the growth arrest specific-1, the neurokinin 1-receptor, as well as the deletion of endothelin receptor type B (*Ednrb*) in a rescued EDNRB knockout mouse enhanced ovulation rate in animals (64–66). Therefore, the recruitment of growing follicles is regulated by multiple negative regulatory pathways, and future studies of these pathways may reveal how *Adm2* disruption in oocytes enhances ovulation rate, and facilitate our ability to improve follicle development in infertile patients.

Our study also showed that *Adm2* disruption reduces the developmental capacity of fertilized eggs *in vitro*. This result could be a consequence of impaired intrafollicular ADM2 signaling. The absence of an ADM2 gradient from oocytes may hinder normal oogenesis. We and others have shown that ADM2 promotes interactions between the oocyte and cumulus cells, and blockage of ADM2 signaling impairs COC formation *in vitro*. Likewise, intrabursal injection of an ADM2 antagonist led to oocyte atresia and disintegration of the COC tertiary structure (38, 39). Furthermore, the present study showed that *Adm2* disruption significantly reduces the size of the corpus luteum in homozygous animals. The corpus luteum is a transient endocrine gland that produces progesterone after ovulation. The rapid growth of the corpus luteum is a result of both proliferation and hypertrophy of luteal cells (67). The reduced corpora luteum size in homozygous animals may be a consequence of inadequate granulosa cell proliferation prior to ovulation in individual follicles. This idea corroborates with the observation that there is a significant reduction of cyclin D2 expression in follicles of homozygous animals and that oocyte quality was reduced in heterozygous and homozygous animals.

The endocrine actions elicited by the superovulation stimulation can lead to complex ovarian differentiation and remodeling processes, which are modulated by pituitary hormones and intraovarian factors. Among the various local factors, recent progress indicated that the epidermal growth factor (EGF) pathway plays a particularly significant role in regulating oocyte maturation and ovulation (68). Following the LH receptor activation, the LH signal was transmitted from the periphery of the follicle to the COC and downregulates the level of 3’5’-cyclic guanine monophosphate while simultaneously providing a meiotic-inducing signal. The EGF system also plays a role in the regulation of amino acid metabolism, and this regulatory pathway may participate in the regulation of competence of COCs and fertility in bovines (69). On the other hand, local angiogenic signals are necessary to provide blood flow to the corpus luteum, thereby allowing it to develop the proper structure and acquire the steroidogenic capacity (70). In ruminants, it was shown that angiogenic factors, including vascular endothelial growth factor-A (VEGFA), insulin-like growth factors, angiopoietins, and fibroblast growth factors, play central roles in promoting cell proliferation, angiogenesis, and blood vessel stability in developing follicle and corpus luteum (71). In addition, studies of the expression of thrombospondins (THBS1 and THBS2) and their receptors (CD36 and CD47) suggested that they may play a role in inhibiting angiogenesis surrounding follicles (72). Furthermore, immune cells that are recruited into the corpus luteum after ovulation may play a role in supporting angiogenesis and the growth of the corpus luteum. Moreover, it has been shown that the lymphatic system is reconstituted in the corpus luteum through lymphangiogenesis in cows during early pregnancy (71, 73). While it is not clear whether ADM2 interacts with these intrafollicular factors in the mouse ovary, the observation that ADM2 deficiency affects follicle development and the size of corpus luteum suggests that ADM2 may participate in the regulation of EGF-mediated COC maturation and angiogenic factor-mediated corpus luteum growth. Because ADM2, like ADM, exhibits potent angiogenic and lymphangiogenic effects in different tissues *in vivo* and affects COC integrity *in vitro*, ADM2 deficiency may reduce angiogenesis and lymphangiogenesis within follicles and corpus luteum, thereby retarding the growth of corpus luteum after superovulation.

It is important to note that decreased hormonal responses are a hallmark of ovarian aging. The recognition that intrafollicular ADM2 signaling plays a role in coordinating follicle growth may provide novel strategies to improve follicle growth in women who exhibit poor ovarian responses after ovarian hyperstimulation in IVF clinics (74, 75). Finally, we also like to note we have recently developed a series of potent agonistic and antagonistic ADM2 analogs (76). Future studies of these analogs on follicle growth and ovulation may provide insights into how CLR/RAMP signaling regulates distinct aspects of ovarian folliculogenesis.

## Conclusions

Disruption of the *Adm2* gene in oocytes significantly increased the number of ovulated oocytes but impaired the egg’s developmental capacity after superovulation. Overall, the study has revealed that oocyte-derived ADM2 plays a facilitative role in the regulation of follicle growth and hormonal responses independent of the closely related ADM and CGRP peptides.

## Data availability statement

The raw data supporting the conclusions of this article will be made available by the authors, without undue reservation.

## Ethics statement

The animal study was reviewed and approved by Chang Gung Memorial Hospital.

## Author contributions

W-CL, T-HL, J-YS, and YS collected, analyzed, and prepared the data and figures. CC conceived, planned, and wrote the manuscript. All authors read and approved the manuscript.

## Funding

This study was supported by Chang Gung Memorial Hospital (CMRPG3C1691-3, CC).

## Acknowledgments

We thank Shu-wha Lin (Department of clinical laboratory sciences and medical biotechnology, National Taiwan University College of Medicine) for technical assistance.

## Conflict of interest

The authors declare that the research was conducted in the absence of any commercial or financial relationships that could be construed as a potential conflict of interest.

## Publisher’s note

All claims expressed in this article are solely those of the authors and do not necessarily represent those of their affiliated organizations, or those of the publisher, the editors and the reviewers. Any product that may be evaluated in this article, or claim that may be made by its manufacturer, is not guaranteed or endorsed by the publisher.

## Supplementary material

Supplementary Figure 1 Identification of wild-type and *Adm2 ^loxP/loxP^* transgenic mice. PCR amplification was used to detect the wild-type and transgene sequences. The 450- and 350-basepair PCR products indicated the presence of transgene and wild-type sequences, respectively. Mouse tail DNA samples in lanes 2, 3, 8, and 10 were from homozygous mice whereas those in lanes 4-7 were from wild-type animals. Samples in lanes 9 and 11-15 were from heterozygous animals. The red horizontal bar indicates the position of the 500-bp size marker.

Supplementary Figure 2 Expression of the wild-type *Adm2* transcript in the ovary and kidney tissues. Reverse transcription-polymerase chain reaction (RT-PCR) was used to detect the presence of wild-type *Adm2* mRNA in the ovary (O) and kidney (K) of wild-type (+/+), heterozygous (+/-), and homozygous (-/-) animals. The 535-basepair PCR product indicates the presence of the wild-typ*e Adm2* mRNA. The primer set sequences used to detect the wild-type *Adm2* transcript are 5’-GAC TGA GGC ACT CCA GAC CCA GAG A-3’ and 5’-TGT CGG TGC AGA TTC TAC AGC CTA GAT-3’. Positions of the *Adm2* and *Gapdh* cDNA products are indicated by arrowheads.
