# Supplementary figures and images for: Oocyte-specific disruption of adrenomedullin 2 gene enhances ovarian follicle growth after superovulation
Source: Front Endocrinol (Lausanne). 2022 Nov 14;13:1047498. doi: 10.3389/fendo.2022.1047498 (PMC9702065; doi:10.3389/fendo.2022.1047498)

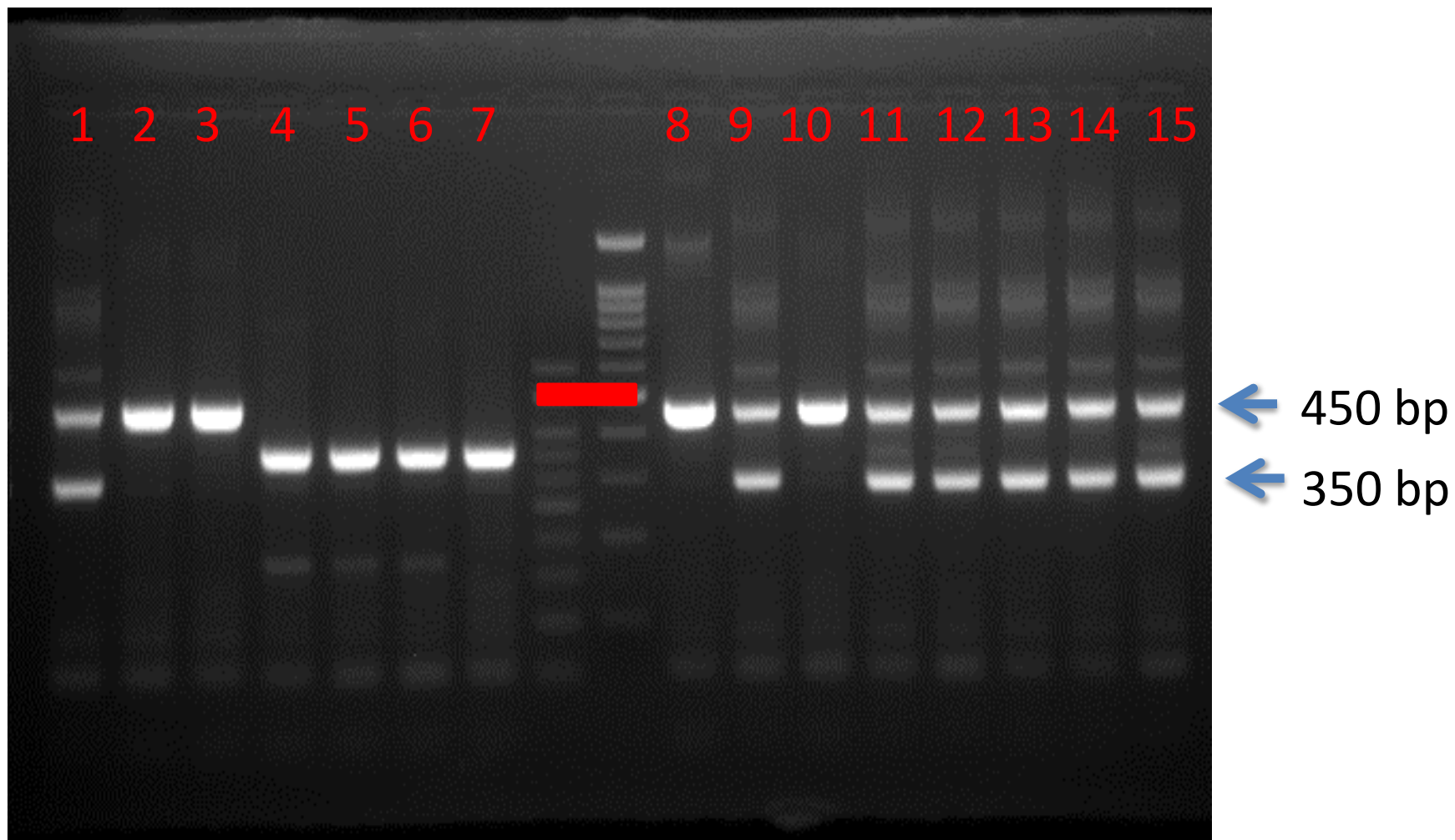

Supplementary Fig. 1

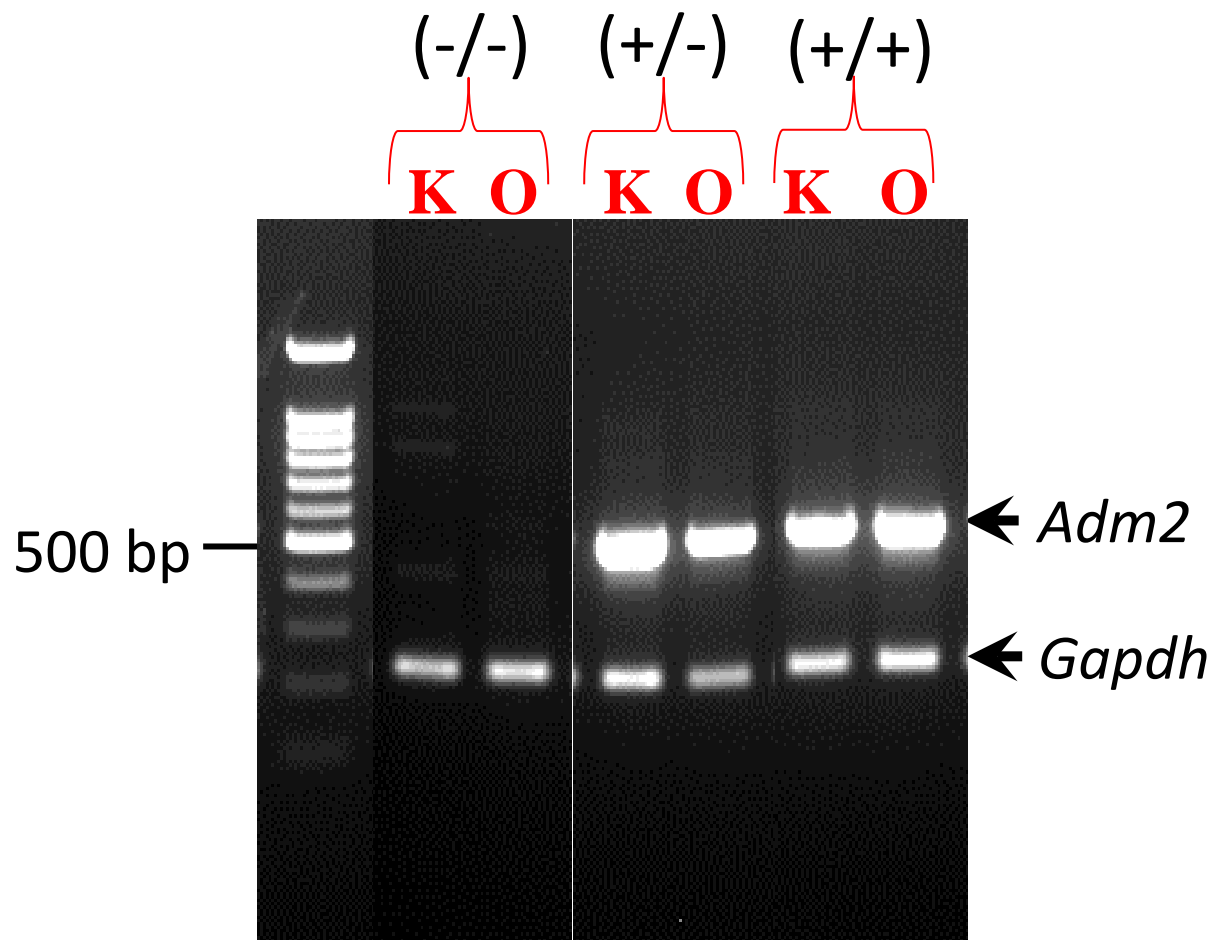

Supplementary Fig. 2

Supplement: Supplementary Figure 1 — Identification of wild-type and Adm2 loxP/loxP transgenic mice. PCR amplification was used to detect the wild-type and transgene sequences. The 450- and 350-basepair PCR products indicated the presence of transgene and wild-type sequences, respectively. Mouse tail DNA samples in lanes 2, 3, 8, and 10 were from homozygous mice whereas those in lanes 4-7 were from wild-type animals. Samples in lanes 9 and 11-15 were from heterozygous animals. The red horizontal bar indicates the position of the 500-bp size marker. [file DataSheet_1.pdf]
